# The Effects of Cancer Immunotherapy on Fertility: Focus on Hematological Malignancies

**DOI:** 10.3390/biomedicines12092106

**Published:** 2024-09-15

**Authors:** Santino Caserta, Gabriella Cancemi, Giuseppe Murdaca, Fabio Stagno, Mario Di Gioacchino, Sebastiano Gangemi, Alessandro Allegra

**Affiliations:** 1 Hematology Unit, Department of Human Pathology in Adulthood and Childhood “Gaetano Barresi”, University of Messina, via Consolare Valeria, 98125 Messina, Italy; santino.caserta@polime.it (S.C.); gabriella.cancemi@polime.it (G.C.); fabio.stragno@unime.it (F.S.); aallegra@unime.it (A.A.); 2 Department of Internal Medicine, University of Genova, 16126 Genova, Italy; 3 Allergology and Clinical Immunology, San Bartolomeo Hospital, 19038 Sarzana, Italy; 4 Institute for Clinical Immunotherapy and Advanced Biological Treatments, 65100 Pescara, Italy; digioacchino@me.com; 5 Center for Advanced Studies and Technology, G. D’Annunzio University, 66100 Chieti, Italy; 6 Department of Clinical and Experimental Medicine, School and Operative Unit of Allergy and Clinical Immunology, University of Messina, 98125 Messina, Italy; gangemis@unime.it

**Keywords:** immunotherapy, immunomodulators, immune checkpoint inhibitors, CAR-T cell therapy, fertility, gonad toxicity, cancer, leukemia, lymphoma, multiple myeloma

## Abstract

In recent years, cancer management has benefitted from new effective treatments, including immunotherapy. While these therapies improve cancer survival rates, they can alter immune responses and cause long-term side effects, of which gonadotoxic effects and the potential impact on male and female fertility are growing concerns. Immunotherapies, such as immune checkpoint inhibitors, immunomodulators, monoclonal antibodies, and CAR-T, can lead to elevated levels of proinflammatory cytokines and immune-related adverse events that may exacerbate fertility problems. Immunotherapy-related inflammation, characterized by cytokine imbalances and the activation of pathways such as AMPK/mTOR, has been implicated in the mechanisms of fertility impairment. In men, hypospermatogenesis and aspermatogenesis have been observed after treatment with immune checkpoint inhibitors, by direct effects on the gonads, particularly through the inhibition of cytotoxic T lymphocyte antigen-4. In women, both damage to ovarian reserves, recurrent pregnancy loss, and implantation failure have been documented, secondary to a complex interplay between immune cells, such as T cells and uterine NK cells. In this review, the impact of immunotherapy on fertility in patients with hematological cancers was analyzed. While this area is still underexplored, fertility preservation methods remain crucial. Future studies should investigate immunotherapy’s effects on fertility and establish standardized preservation protocols.

## 1. Introduction

The long-term side effects of cancer treatment continue to affect a significant portion of the population, despite the increased survival rate among cancer patients. One of the most perilous adverse effects is the off-target impact on germ cells, leading to the depletion of primordial follicles within the ovarian reserve [1]. The ovarian reserve is defined as the quantity and quality of follicles in the ovary that remain accessible for future fertility and endocrine support for women. A severe consequence of this is Primary Ovarian Insufficiency (POI), which exerts a profound negative influence on women’s physical and mental health. Clinically, POI manifests as endocrine malfunction and infertility [1,2].

### 1.1. Immunological Background

The immune system, particularly T cells and uterine NK (uNK) cells—a unique subpopulation found in the uterus—has been demonstrated to regulate processes crucial for successful implantation and pregnancy maintenance, promoting maternal tolerance [3,4].

In efforts to identify potential immunological dysregulation, researchers examined the immune cell composition of the endometrium in patients experiencing recurrent pregnancy loss (RPL) and recurrent implantation failure (RIF) [5]. Patients with RPL exhibited elevated levels of cytotoxic CD56dimCD16, while those with RIF showed an increased presence of CD56+ uNK cells [1,5,6,7,8,9,10]. For a considerable amount of time, the division of CD56dim NK cells with cytotoxic activity and CD56bright NK cells with cytokine production has dominated the literature. However, recent research suggests a more diverse categorization of NK cells, based on the expression levels of CD56 and CD16 [11].

It has been established that women with RPL are more likely to possess a specific subset of NK cells (CD56dimCD16high) compared to those with RIF, despite numerous studies demonstrating increased levels of CD56+ uNK cells. Moreover, the finding that patients with RPL and pregnancy complications exhibit a higher percentage of NKT cells is intriguing [10,11]. NKT cells represent a distinctive subset of T cells with both T cell and NK receptors, playing a crucial role in immune regulation, inflammation, and tissue repair in various physiological and pathological conditions [12]. The key elements for a successful pregnancy are the mother’s immunological tolerance mechanisms against an antigenically alien fetus without a corresponding loss of defense capacities against infections.

In this context, the immune response is crucially related to the cellular microenvironment in which exosomes can be found: They are endovesicles with a 40–100 nm diameter that facilitate intercellular communication through the transfer of genetic material like microRNAs (miRNAs), lipids, and proteins. In detail, it has been demonstrated that adipose-derived stem cell exosomes (ADSCs-Exo) can enhance autophagy and mitigate podocyte damage by preventing the activation of the AMPK/mTOR pathway [13,14,15]. Moreover, as shown, the release of GATA3 from exosomes derived from tumor-associated macrophages has been shown to promote tumor growth in the tumor microenvironment of high-grade serous ovarian carcinoma [16].

Adipose-derived stem cells (ADSCs), originating from adipose tissue, possess the unique ability to differentiate into various cell types and tissues under specific induction conditions. ADSCs-Exo, vesicles released by ADSCs, play a crucial role in mediating these functions. In a groundbreaking discovery, researchers have revealed that exosomes derived from bone marrow mesenchymal stem cells can protect rats against Primary Ovarian Insufficiency (POI). The AMPK/mTOR pathway has been shown to control oocyte aging and female reproduction. It also plays a crucial role in the regulation of cell autophagy, which may contribute to the development of premature ovarian failure (POF). A member of the metabolite-sensing protein kinase family, AMPK can obstruct mTOR activation, which, in turn, plays a role in fertility’s metabolic gating. The dysregulation of mTOR signaling, characterized by its overexpression, may lead to insulin resistance, disruption of follicle growth, and interference with cumulus cell interaction—all potential contributors to POF. For example, in ovarian germ cells (GCs), bisphenol A stimulates the AMPK/mTOR signaling pathway and encourages autophagy [17,18,19,20]. Similarly, research indicates that nonylphenol stimulates the rat ovaries’ GCs to undergo both autophagy and apoptosis. This phenomenon may be connected to the AMPK/mTOR pathway’s AMPK/ROS-dependent activity. As medically assisted reproductive technologies, including oocyte cryopreservation, continue to advance, more options for preserving fertility are being made available [17,21,22].

Killer Immunoglobulin-like Receptors (KIRs) play a pivotal role in human defense by regulating the activity of Natural Killer (NK) cells. These cells are essential for eliminating antigenically foreign cells, virus-infected cells, and tumor-lesioned cells. Immunological infertility, characterized by a woman’s inability to conceive or sustain a pregnancy due to immune system attacks on her own reproductive cells, is often attributed to immune system disruption or the overactivity of immune-competent cells. The inflammatory response, involving NK cells and KIR receptors, is hypothesized to significantly contribute to infertility. Major Histocompatibility Complex (MHC) molecules, particularly Human Leukocyte Antigen (HLA), are crucial for successful pregnancy [23,24]. During embryo implantation, the interaction between KIR receptors on female uterine NK cells and HLA class I molecules, with HLA-C playing a key role on the surface of germ cells, becomes strategically important. In addition, the healthy development of the placenta and successful embryo implantation in the uterus rely on maintaining a functional balance between activating and inhibitory KIR receptors.

### 1.2. Immunotherapy-Related Inflammation: A Systemic Cytokine Imbalance

Pregnancy problems may arise from an imbalance in this system brought on by the use of immunotherapies [25,26,27,28,29,30]: As a matter of fact, even though these drugs are being tested more and more in the frontline setting for pediatric cancers, their current approved use in prepubertal girls and women of reproductive age may pose an additional gonadotoxic burden as a result of chemotherapy and radiation therapy. As a result, there is a good chance that changes in circulating immune cells brought about by immunotherapy may have an immediate effect on ovarian follicles, as evidenced by the depletion of primordial follicles and the infiltration of ovarian CD3+ T cells after injections of pembrolizumab and anti-mouse PD-1 antibody in immunocompetent mice.

The connection between immunotherapy-induced inflammation and reproductive health is an emerging research area. Chronic inflammation is known to negatively impact various aspects of fertility. Immunotherapy, designed to activate the immune system against cancer, may potentially amplify these inflammatory responses. While this inflammation can be effective against tumors, it may also have harmful side effects on healthy tissues, including reproductive organs. Specifically, inflammation can alter the ovarian and uterine microenvironment, with uterine inflammation linked to reduced embryo implantation and ovarian inflammation potentially accelerating ovarian aging and reducing ovarian reserve, crucial for long-term fertility [31].

**Figure 1:**
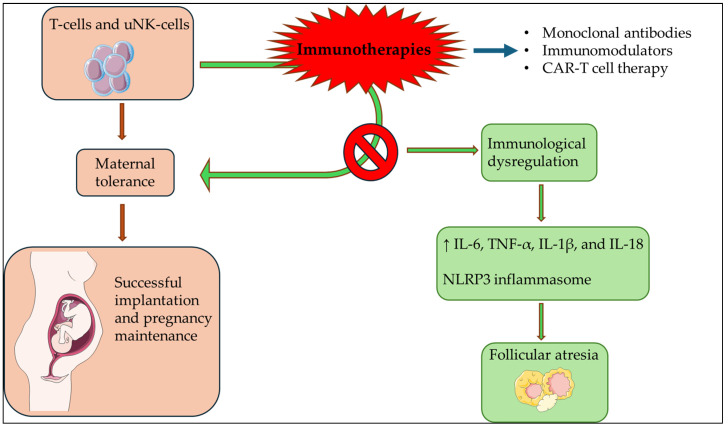
Immunotherapies induce immunological dysregulation, resulting in follicular atresia. The Figure was partly generated using Servier Medical Art, provided by Servier, licensed under a Creative Commons Attribution 3.0 unported license.

Pro-inflammatory cytokines, including IL-6, TNF-α, IL-1β, and IL-18, are often elevated in chronic, sterile, low-grade inflammation. This type of inflammation is commonly characterized by the activation of the NLRP3 inflammasome in innate immune cells like macrophages, leading to the mediation of follicular atresia. In the context of ovarian aging, recent research indicates that persistent low-grade systemic and local inflammation, mediated by the NLRP3 inflammasome, contributes to ovarian follicle depletion. Studies have demonstrated that treatment with an anti-mouse PD-1 antibody results in the upregulation of pro-inflammatory cytokines TNF-α and COX-2, an enzyme primarily responsible for generating inflammation [32,33,34] (Figure 1).

Additionally, checkpoint inhibitors have been shown to be the immunomodulators most strongly linked to adverse effects on the endocrine system, such as hypophysitis and hypothyroidism. Specifically, it has been shown that 1% to 11% of women on PD-1/PD-L1 inhibitors and up to 11% of those taking CTLA-4 inhibitors get hypophysitis, while 6% of women taking PD-1/PD-L1 inhibitors and 15% of those using CTLA-4 inhibitors report having hypothyroidism [35,36,37].

Regarding male fertility, Salzmann et al. examined the sperm of 25 patients receiving checkpoint inhibitor treatment. Out of them, three had azoospermia, one had oligoasthenoteratozoospermia, and 82% had normal semen analysis. However, there were important confounding factors (previous inguinal irradiation, chemotherapy, chronic alcohol misuse, and bacterial orchitis) in three of the individuals with aberrant semen results. Conversely, one patient with a normal semen analysis before treatment developed azoospermia with an inflammatory infiltrate predominantly composed of neutrophils, macrophages, and T lymphocytes one year after therapy. These results imply that primary hypogonadism in men may be an uncommon side effect of immunotherapy [38].

However, the exact mechanisms by which immunotherapy-induced inflammation affects fertility are not yet fully understood. Ongoing research is exploring these dynamics with the aim of developing strategies to mitigate these undesirable effects without compromising the antitumor efficacy of immunotherapy.

## 2. Immunotherapy: A Tailored Treatment for Cancer

Over the past few decades, advances in genetic sequencing, cell signaling, and cancer biology have changed the therapy of human malignancy. More precisely, they have facilitated the switch from conventional cytotoxic chemotherapy to small molecule cell-signaling inhibitors and tailored immunotherapy. These targeted medicines have several advantages, including better survival rates and tailored treatment plans. The two classes of targeted cancer therapy are small molecule inhibitors and immunotherapy together.

Interferon-alpha 2 was the antitumor cytokine that became the first immunotherapy to receive FDA approval in 1986 [39,40,41]. Since then, adoptive cell therapy, monoclonal antibodies, immunomodulators, and therapeutic vaccines have emerged as the four main subclasses of immunotherapy, with differences in their mechanisms of action. Since imatinib’s approval in 2001, small molecule inhibitors—which alter cell-signaling pathways crucial for tumor growth—have also distinguished themselves into four distinct subclasses: metalloproteinases and heat shock protein inhibitors, promoters of apoptosis, kinase inhibitors, and proteasome inhibitors [40].

### 2.1. Mechanisms of Gonadotoxicity

#### 2.1.1. Aging and Traditional Drug-Related Gonadotoxicity

In the human female reproductive system, immunity only becomes apparent during pregnancy and semen exposure. Ovulation, atresia, and folliculogenesis all depend on circulating immune cells and cytokines, which can penetrate the ovaries due to its well-vascularized structure and resident immune cells. These cytokines can be produced by non-immune cells like follicular and oocyte cells, or by the immune cells that are present [31,42,43,44,45,46,47]. Ovarian reserve can be significantly lost during chemotherapy treatments, and as ovarian reserve declines with age, older women are more susceptible to POI. Researchers found that women under 35 had a 15–30% likelihood of developing POI, whereas those over 40 had a 50% chance.

One noteworthy observation about ovarian aging is that oocyte quality also declines with aging, in addition to oocyte quantity. Permanent harm to the ovaries due to primordial follicle depletion or death, vascular injury, fibrosis, or stromal damage is the cause of long-term consequences. First, either rapid recruitment or direct DNA changes might result in the loss of primordial follicles.

Primordial follicle apoptosis can result from DNA modifications and double-strand breaks caused by alkylating chemicals and anthracyclines. Nonetheless, some follicles will be able to survive and repair damage to deoxyribonucleic acid (DNA). Another theory that has been proposed to explain primordial follicle depletion contends that chemotherapy causes a “burn-out” effect by hastening the recruitment of dormant follicles in an effort to replenish the damaged follicles [48]. When further chemotherapy is given, primordial follicles mature into more delicate primary or secondary follicles, dealing the ovaries a second blow. The activation of the phosphoinositide 3-kinase (PI3K) signaling pathway, which has a well-established role in follicle quiescence, would induce the recruitment of primordial follicles. This pathway is activated in response to a decrease in the anti-Müllerian hormone [AMH] secretion that occurs after follicle apoptosis [44,49,50].

#### 2.1.2. New Immunotherapeutic Agents’ Involvement in Gonadotoxicity Mechanisms

In the last few decades, targeted therapies based on the molecular and genetic makeup of the tumor have become a part of cancer treatment. These therapies have not only increased the overall survival rate of cancer patients, but they also typically cause fewer side effects than traditional chemotherapy. To be more specific, the development of immune checkpoint inhibitors (ICIs) as a form of immunotherapy has increased the survival rate of cancer patients in both adjuvant and metastatic situations. ICIs, or immune checkpoint inhibitors, are drugs that block immune checkpoint proteins, such as cytotoxic T-lymphocyte-associated protein 4 (CTLA4), planned death ligand-1 (PDL1), and programmed death-1 (PD1); currently, they are part of the conventional treatment for a number of cancers, including melanoma and multiple myeloma [51,52]. The antitumor immune response is triggered and strengthened by the immune system being allowed to loosen its grip. Complete responses are recorded for ICIs in the metastatic setting, and in the adjuvant setting, they dramatically lower the chance of relapse. There is currently very little information on the impact of ICIs on fertility, despite the fast development of their indications for use. Immune-related adverse events (irAEs) are a part of ICI treatment and can impact almost any organ, including the endocrine system. Adverse conditions linked to the endocrine system, such as hypophysitis and hypothyroidism, have been well-documented and have the potential to cause ovarian insufficiency by decreasing gonadotropin output. Scientists describe the hypothesis that immunotherapy may affect fertility by altering circulating immune cells, such as mast cells, T-lymphocytes, neutrophils, and macrophages, and elevating cytokines, such as interleukin 1 and tumor necrosis factor α, even though the mechanisms underlying this are not fully understood [53,54,55]. While women treated with ICIs can develop hypophysitis, there is evidence of orchitis associated with primary hypogonadism in men: In detail, it has been observed that anti-PD1 and anti-CTLA4 are associated with reduced spermatogenesis and cause partial inhibition in the synthesis of testosterone. However, the onset of these negative consequences on fertility is not immediate; in fact, normo-zoospermic men treated with anti-PD1 and anti-CTLA4 experience azoospermia after more than two years of therapy. Unfortunately, the majority of these endocrine adverse effects are irreversible [56] (Table 1).

### 2.2. Monoclonal Antibodies

The impact of monoclonal antibodies (mAbs) on ovarian function appears to be contingent upon the target of interest and the stage of female sexual development at which exposure to therapy takes place. Many of the cell-surface receptors that are frequently targeted by monoclonal antibodies have been identified by the Genotype-Tissue Expression Portal as receptors that are also highly expressed in normal ovarian tissue.

One such receptor that has been repeatedly identified as necessary for healthy ovarian and follicular development is the Vascular Endothelial Growth Factor Receptor (VEGF-R). It is particularly concerning for young women who have not yet reached puberty, as the suppression of VEGF-R could impair ovarian function in the future irreversibly. It is interesting to note that research on women whose ovaries have already reached their full developmental stage indicates that mAbs may have a neutral or even positive effect [57].

In particular, adding trastuzumab, an inhibitor of the receptor tyrosine–protein kinase Human Epidermal Growth Factor Receptor 2 (HER2) used in breast, gastric, and non-small cell lung cancer, to the treatment regimen had no effect on the serum anti-Müllerian hormone (AMH) level in premenopausal women (aged 25–50) who received chemotherapy for breast cancer. Rather, trastuzumab exposure was linked to an overall rise in AMH levels in survivors who reported regular menstruation while receiving treatment. This suggests that adjuvant trastuzumab may protect ovarian reserve, and it is consistent with two other studies that have been published [58,59,60,61]. Trastuzumab’s safety for ovarian function is further supported by data from the ALTTO study [NCT00490139], which showed that adding trastuzumab to a treatment combination of lapatinib and cytotoxic chemotherapy did not raise the incidence of treatment-related amenorrhea in breast cancer patients. Future research is required to assess the results of using MAbs as a single agent as opposed to in combination with cytotoxic chemotherapy [43,54].

A special mention must be made of Antibody–Drug Conjugates (ADCs), a class of targeted oncology drugs in which a monoclonal antibody is attached to a cytotoxic drug via a linker. These drugs, although directed primarily towards tumor cells, can have systemic side effects, albeit reduced compared to classic chemotherapeutics, including damage to reproductive tissues [62,63]. Maristany et al. described a case of rapid-onset POI in a 23-year-old patient with relapsed/refractory B-cell acute lymphoblastic leukemia after therapy with inotuzumab ozogamicin, an ACD targeting CD22 [64]. On the other hand, a case of successful pregnancy after treatment with brentuximab vedotin in a patient with classical Hodgkin lymphoma has been described [65]. Therefore, the impact of ADCs on fertility is not yet fully understood, and information in this field is limited.

### 2.3. Immunomodulators

As a subclass of immunotherapy, immunomodulators alter important cell-signaling pathways to boost the innate and adaptive immune systems’ response and enhance the immune system’s capacity to identify tumors as non-self entities. These substances fall into different categories, including checkpoint inhibitors, adjuvant treatments, and cytokines. Cytokines are proteins that use autocrine and paracrine signaling pathways to help the immune system grow and mature locally; adjuvants further activate the innate and adaptive immune system, especially dendritic cells, by activating cell pattern recognition receptors, which increases the transcription of critical signaling pathways like NF-κB and interferon. Checkpoint inhibitors, as previously mentioned, block the cell-signaling pathways that tumor cells use to suppress the activation of immune cells by preventing checkpoint proteins on the surfaces of tumor cells from connecting with partner proteins on nearby leukocytes [66,67].

#### Immune Checkpoint Inhibitors

Checkpoint inhibitors are the most strongly linked of the three immunomodulator classes to adverse effects on the endocrine system; pituitary inflammation, or hypophysitis, and hypothyroidism are the two most prevalent manifestations: While hypothyroidism (all grades) is observed in 6% of women using PD-1/PD-L1 inhibitors and 15% of those using CTLA-4 inhibitors, hypophysitis has been reported to occur in 1–3% of women getting PD-1/PD-L1 inhibitors and up to 11% of those receiving CTLA-4 inhibitors [43,68,69,70,71,72,73].

Immune-related adverse events (irAEs) are specific inflammatory side effects linked to immune checkpoint inhibitors [74]. The impact of immune checkpoint inhibitors on fertility and subsequent pregnancies is a persistent issue associated with their use; in fact, anti-PD-1 agents may have direct effects on oogenesis and follicular development.

The majority of studies conducted in preclinical and clinical research are overwhelmingly focused on measuring tumor regression and finding biomarkers that indicate response to immunotherapy treatment. Regrettably, no clinical trials pertaining to reproduction or fertility were carried out before the FDA approved all of the immune checkpoint inhibitors that are currently on the market.

Pembrolizumab, a humanized IgG4 monoclonal antibody, is presently approved for the treatment of children with relapsed or refractory classic Hodgkin lymphoma, recurrent or locally advanced or metastatic Merkel cell carcinoma, metastatic and unresectable microsatellite instability-high or mismatch repair deficient cancer, and refractory or relapsed primary mediastinal large B-cell lymphoma [75,76,77].

Preclinical evidence in non-human primates after one month and six months showed no significant effects on the female or male reproductive organs, despite the fact that no human trials have been conducted to assess the possible genotoxicity of pembrolizumab [78,79].

An intriguing study reported high tracer uptake and high estimated radiation-absorbed doses to adult humans in the ovaries using positron emission tomography (PET) imaging to map the biodistribution of pembrolizumab radiolabeled with the positron-emitting radionuclide 89Zr over 168 h (7 days) in two normal rodent models and a humanized mouse model implanted with human peripheral blood mononuclear cells [31]. According to data from the World Health Organization’s (WHO’s) spontaneous reporting system, 56 patients reported pregnancy-related outcomes, the most common of which were diarrhea, nausea, fatigue, abdominal pain, pruritus, and chest pain; the most common fetal outcomes, on the other hand, were spontaneous abortions and prematurity.

Current guidelines advise against using ICIs in patients of childbearing age (unless they are using effective contraception) during and for at least five months after the last dose of ICI treatment. Endocrine toxicities, such as immune-related hypothyroidism or hyperthyroidism, hypophysitis, adrenal insufficiency, and type 1 diabetes mellitus, can result in irreversible organ damage requiring lifelong hormonal supplementation. There is a higher incidence of endocrine immune-related adverse events with CTLA-4 blockade than with PD-1, and a higher incidence of hypothyroidism with PD-1/CTLA-4 blockade compared to CTLA-4 monotherapy [80,81,82,83,84,85]. These toxicities can cause life-threatening inflammation in almost any organ system. Furthermore, gonadal toxicity, including libido, erectile function, and sexual quality of life for men and women, has not always been reported in ICI therapy trials across disease types [86]. In a small series of men with melanoma who received ICI treatment, testicular histopathology revealed that more than 80% had hypospermatogenesis or aspermatogenesis [59].

### 2.4. CAR-T Cell Therapy

Numerous distinct CAR T-cell constructs are approved for use in treating a variety of relapsed/refractory B-cell malignancies in patients of all ages. Chimeric antigen receptor (CAR) T-cell-based immunotherapeutic strategies have proven to be highly effective in treating relapsed/refractory B-cell malignancies. Acute toxicities associated with CAR T-cell growth are well-established and include a systemic inflammatory response, also known as cytokine release syndrome and its sequelae, which is based on using the immune system to drive tumor death. However, long-term monitoring is still being conducted because the effects of CAR T-cells are not fully understood. Immunotherapy is thought to prevent long-term toxicities linked to cytotoxic treatments, like infertility, which is one possible benefit [43,87,88,89].

For both men and women receiving cancer therapy, fertility is a significant worry; this is especially true for children and adolescents/young adults (AYAs), who still have the majority of their reproductive years ahead of them. More focus has been placed on providing patients with cancer with options for fertility preservation, such as oocyte cryopreservation, ovarian tissue cryopreservation, and sperm cryopreservation, in order to improve their quality of life after treatment, since over 80% of children and AYAs with cancer are expected to survive for longer than five years.

Due to the fact that cumulative exposure to alkylating chemotherapy, radiation, and hematopoietic stem cell transplantation (HSCT) has been shown to be a primary risk factor for infertility after cancer therapy, fertility preservation before therapy, or earlier in the course of treatment, is preferred. Because most patients treated with CAR T-cells have many relapses or are resistant to treatment, worries about fertility have not been at the forefront of this emerging field. Additionally, the entire potential of these cells remains undiscovered. However, as CAR T-cell therapy progresses, prospective studies are imperative to precisely ascertain the impact of these genetically modified T-cells on gonadal function, since they may endure over time and potentially minimize exposure to gonadotoxic therapies early in the treatment plan [90,91].

In addition to gathering data on early experiences with post-CAR T-cell fertility and gonadal function, researchers aimed to identify practice patterns in approaches to fertility and fertility maintenance in the peri-CAR T-cell period: Six centers (9.8%) out of sixty-one respondents reported seven total pregnancies (four female patients who conceived and three male patients whose partners conceived). Of these, five confirmed live births following CAR T-cell therapy, and two with uncertain outcomes were reported; no pregnancy complications or spontaneous or elective abortions were reported. Following CAR T-cell therapy, seven centers reported referring thirteen patients for assisted reproductive techniques. Of those patients, two pregnancies were reported as outcomes; the same center also reported using donor sperm and eggs, but no successful pregnancies were reported. Patients at two facilities reported beginning a family through surrogacy or adoption in addition to traditional methods. Crucially, the majority of CAR T-cell clinics have initiatives in place to preserve fertility. This pre-existing infrastructure creates a chance to work together on this new area of onco-fertility issues and gives our immunotherapy providers and our patients undergoing CAR T-cell therapy access to specialists in fertility preservation. Future work should involve the natural development of peri-CAR T-cell guidelines, the monitoring of fertility measures, and consultation with pediatric gynecology, urology, and reproductive endocrinology and infertility.

The following should be taken into account while discussing when to preserve fertility: Consideration should be given to post-CAR T-cell fertility preservation prior to myeloablative hematopoietic stem cell transplantation (HSCT) in patients who may be using CAR T-cells for remission induction prior to more intensive therapies [92,93,94,95]. This may be an advantageous time to leverage a remission status while preparing for HSCT. Furthermore, given the necessity to begin therapy immediately and the possibility of ovarian tissue involvement due to cancer, which may be eliminated by CAR T-cells, given their capacity to eliminate extramedullary diseases, this may be especially crucial for patients with hematologic malignancies, who frequently do not have the chance to undergo initial pretreatment fertility preservation [56].

## 3. Preservation of Fertility

Having children after receiving a cancer diagnosis is crucial for the psychological health of many women. On its own, infertility is associated with significant psychological discomfort and double the rate of depression compared to the general population. Furthermore, this results in a decline in life quality with regard to relationships, sexuality, and emotional health [96,97]. Despite that, according to a study by Schover and colleagues, 76% of cancer patients who were of childbearing age wanted to start a family once their cancer had healed, with the likelihood of infertility increasing with the kind and age of cancer therapy [98].

Therefore, it makes sense to discuss these possibilities with patients after delivery and to have their reproductive capacity assessed. The gonadotoxic effects of radiation and chemotherapy are well-established; on the other hand, studies on the possible gonadotoxicity of anti-PD-1, anti-CTLA-4, anti-LAG3, and other ICI therapies are just now beginning. The literature that reports high rates of azoospermia and hypospermia highlights the possibility that both men and women could be impacted by these negative consequences. Counseling patients on family planning following therapy and fertility preservation prior to treatment is, therefore, still difficult. Patients are classified into three groups based on their likelihood of becoming pregnant after anticancer treatment: low (30%), medium (30–70%), and high (70%) risk [99]. It is important to emphasize that side effects from medium-risk medical treatments, like immunotherapy, might not show up clinically for several months after the treatment is finished [21] (Table 2).

**Figure 2:**
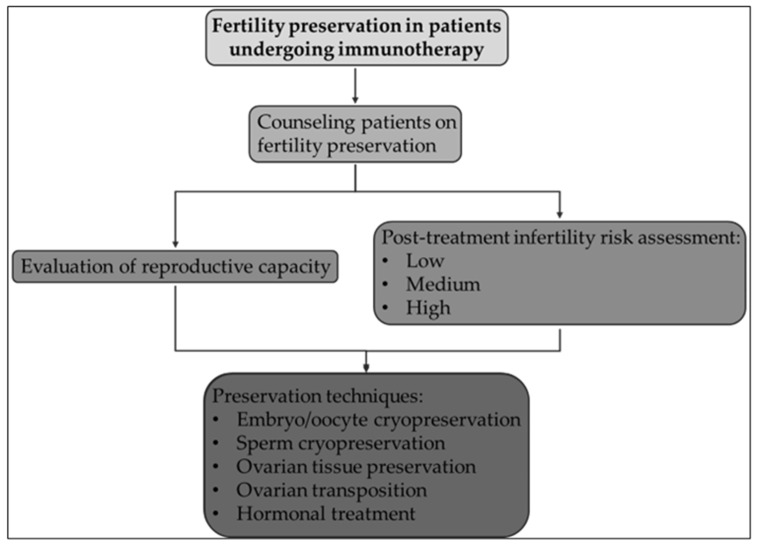
Approach to the patient undergoing immunotherapy at childbearing age.

Embryo/oocyte cryopreservation and sperm cryopreservation, for female and male patients, respectively, are the gold-standard treatments for fertility preservation. Cryopreservation techniques are primarily utilized to preserve the possibility of conception in patients with cancer who are of reproductive age (Figure 2). In order to cryopreserve embryos and oocytes, patients must undergo controlled ovarian hyperstimulation with gonadotropins, which is followed by oocyte retrieval. Cycles of stimulation can be expensive and time-consuming. Random-start controlled ovarian stimulation (COS) methods, on the other hand, shorten the length of a cycle, reducing the amount of time before cancer therapy. Another choice is the cryopreservation of glandular tissue, such as ovarian tissue. This method works best on prepubertal female patients, although it still involves surgery and is still deemed experimental. It is advised that women utilize barrier contraception while on immunocompetent chemotherapy (ICI), and that males should wait two months or six months following treatment completion, before attempting to conceive; moreover, a recommendation for a fertility specialist might be necessary [100,101,102].

The development of clinically useful maternal and fetal cytokine and immune biomarker panels that can assist in determining fetal risk and the status of feto-maternal immune tolerance is urgently needed because it is evident that feto-maternal immune tolerance events require precise temporal drive and regulation for a successful term pregnancy. These panels could provide our ICI patients with options for a precise, logical, and “immunologically tuned” strategy to preserve feto-maternal tolerance. In this situation, patients with cancer can have their reproductive requirements met by interdisciplinary onco-fertility teams that include oncology, reproductive endocrinology, and high-risk maternal and fetal medicine [59].

## 4. Conclusions and Future Perspectives

Immunotherapies and fertility research should focus on crucial themes: (1) to gather long-term data on fertility following immunotherapy; (2) to understand the impact of immunotherapies on a growing fetus; and (3) to establish consistent fertility guidance in the peri-immunotherapy scenario. Immunotherapy-induced persistent inflammatory activity has been seen following immunotherapy withdrawal in large arteries and synovial fluid, as well as the primary tumor sites [103,104,105,106]. More specifically, the activation of T-cells associated with extended exposure to proinflammatory cytokines like TNF-α is thought to contribute to the development of chronic inflammatory diseases like rheumatoid arthritis, and TNF inhibition has been proposed to enhance the anti-tumor effect of immune checkpoint inhibitor therapy while concurrently mitigating immune-mediated adverse events.

Cancer patients frequently undergo repeated immunological checkpoint inhibitor treatment cycles; therefore, the potential for abnormal puberty and reduced reproductive lifespan due to chronic inflammatory activity cannot be ignored and should be taken into consideration in future studies [107,108,109,110,111]. Although, significant effects on ovulation and the onset of puberty following two treatment cycles of mouse anti-mouse PD-1 antibody have not been demonstrated [31,112,113]. In fact, therapeutically relevant immunotherapies targeting PD-1, LAG-3, and TIM-3 were found to have no effect on hormonal balance or the quantity or quality of ovarian follicles in mouse models. In conclusion, these scientific findings point to immunotherapy as a potentially useful part of fertility-preserving treatment plans for patients hoping to maintain their ovarian and endocrine function following cancer treatment [114].

## Figures and Tables

**Table 1:** Immunotherapies and their mechanisms of gonadotoxicity.

Immunotherapeutic Agent	Mechanism	Reference
Monoclonal antibodies	Binding to receptors highly expressed in normal ovarian tissue, such as VEGF-R.	[54]
Immunomodulators, in general	Action on the HPO axis, decreased secretion of prostaglandin F2 alpha and oxytocin; development of autoimmune diseases, hypophysitis, and hypothyroidism.	[39]
Immune checkpoint inhibitors, in detail	Endocrine toxicities, hypospermatogenesis, and aspermatogenesis; high absorption of anti PD-1 at the ovarian level.	[55] [31]
CAR-T cell therapy	If present, not fully understood.	[56]

**Table 2:** Risk of permanent infertility according to age and treatment.

Grade of Risk	% Permanent Infertility	Drugs
High	>80%	Radiotherapy on ovaries and testicles; CMF [cyclophosphamide, methotrexate, 5-fluorouracil] in over-40 patients; BC (busulfan, cyclophosphamide)
Intermediate	20–80%	Immunotherapeutic agents; CMF (cyclophosphamide, methotrexate, 5-fluorouracil) in under-40 patients;
Low	<20%	ABVD (adriamycin, bleomycin, vinblastine, dacarbazine); CHOP (cyclophosphamide, vincristine, doxorubicin, prednisone)

## Data Availability

Not applicable.
